# Regulation of membrane fatty acid composition by temperature in mutants of Arabidopsis with alterations in membrane lipid composition

**DOI:** 10.1186/1471-2229-4-17

**Published:** 2004-09-17

**Authors:** Deane L Falcone, Joseph P Ogas, Chris R Somerville

**Affiliations:** 1 Department of Agronomy and the Kentucky Tobacco Research & Development Center, University of Kentucky, Lexington, KY 40546 USA; 2 Department of Biochemistry, Purdue University, W. Lafayette, IN 47907 USA; 3 Carnegie Institution, Department of Plant Biology, 260 Panama Street, Stanford, CA 94305 USA; 4 Current Address:Department of Biological Sciences, University of Massachusetts Lowells One University Avenue, Lowell, MA/01854 USA

**Keywords:** Membrane ipids, thermotolerance, fatty acid desaturase, glycerolipid pathway, PS II fluorescence

## Abstract

**Background:**

A wide range of cellular responses occur when plants are exposed to elevated temperature, including adjustments in the unsaturation level of membrane fatty acids. Although membrane bound desaturase enzymes mediate these adjustments, it is unknown how they are regulated to achieve these specific membrane compositions. Furthermore, the precise roles that different membrane fatty acid compositions play in photosynthesis are only beginning to be understood. To explore the regulation of the membrane composition and photosynthetic function in response to temperature, we examined the effect of temperature in a collection of mutants with altered membrane lipid fatty acid composition.

**Results:**

In agreement with previous studies in other species, the level of unsaturation of membrane fatty acids in Arabidopsis was inversely correlated with growth temperature. The time required for the membrane fatty acids to attain the composition observed at elevated temperature was consistent with the timing required for the synthesis of new fatty acids. Comparisons of temperature-induced fatty acid alterations in membranes were made among several Arabidopsis lines including wild-type Columbia, and the compositional mutants, *fad5*, *fad6*, *act1 *and double mutants, *fad7 fad8 *and *act1 fad6*. The results revealed key changes that occur in response to elevated temperature regardless of the specific mutations in the glycerolipid pathway, including marked decreases in trienoic fatty acids and consistent increases in unsaturated 16:0 and in dienoic 18:2 levels. Fluorescence measurements of various mutants indicated that photosynthetic stability as well as whole plant growth at elevated temperature is influenced by certain membrane fatty acid compositions.

**Conclusions:**

The results of this study support the premise that defined proportions of saturated and unsaturated fatty acids in membrane lipids are required for photosynthetic thermostability and acclimation to elevated temperature. The results also suggest that changes in the membrane fatty acid composition brought about in response to temperature are regulated in such a way so as to achieve highly similar unsaturation levels despite mutations that alter the membrane composition prior to a high-temperature exposure. The results from examination of the mutant lines also suggest that interorganellar transfer of fatty acids are involved in mediating temperature-induced membrane alterations, and reveal steps in the fatty acid unsaturation pathway that appear to have key roles in the acclimatization of membranes to high temperature.

## Background

One of the most prevalent environmental challenges encountered by plants is the exposure to a broad range of temperatures. Plants use a variety of anatomical, metabolic and cellular strategies to deal with changing environmental temperatures. Acclimation to elevated temperatures is mediated at the cellular level in part by the induction of general stress responses, which include the increased expression and activity of heat shock proteins [1-6]. These proteins enable organisms to withstand elevated temperatures by functioning as molecular chaperones to offset damage from misfolded proteins that would otherwise accrue during exposure to high temperature. Changes in the properties of cellular membranes, on the other hand, occur to ensure the proper function of processes that take place within them. The chloroplast thylakoids house the photosynthetic electron transport machinery, and are the most abundant membranes of leaf tissue. They are responsible for all light harvesting and photosynthetic energy conversion in the cell. Some alterations within thylakoid membranes occur rapidly to diminish stress triggered by immediate changes in the environment. For example, short-lived alterations in membranes can take place to counter excess absorbed energy in response to sudden exposures to high light [7,8]. Thermal dissipation of such excess light energy is mediated by the function of the carotenoid zeaxanthin, which is converted from violaxanthin by a de-epoxidase that is rapidly activated in response to high light [9]. In general, however, temperature-induced compositional changes of membranes follow time scales that reflect the acclimatization of plants at different temperatures [10].

Early studies of the ability of plants to acclimate to higher temperatures were conducted on plants adapted to high temperature growth [10-12]. For example, the desert shrub *Atriplex lentiformis*, changes its membrane fatty acid composition by decreasing the level of unsaturated fatty acids such as hexadecatrienoic acid (16:3) and increasing the level of saturated lipids at higher growth temperatures [13]. Membrane fatty acids of plants from temperate environments show similar trends in response to temperature, an observation that suggests that alterations in membrane lipids generally contribute to the ability of plants to acclimate to different temperatures [14,15].

Of the numerous cellular processes likely to be affected by the membrane composition, photosynthesis is probably the most critical. Adjustments in the level of unsaturation of thylakoid membranes may therefore affect the capacity of plants to adapt to elevated temperature conditions in order to avoid a reduction in photosynthetic efficiency. In this regard, the reaction center of PSII is considered to be the most sensitive component of the thylakoid membrane to thermal breakdown, and the function of the water-splitting D1 protein within PSII has been implicated as the most readily damaged by high temperature [10] as well as the being the primary target for photoinhibition [16,17]. High-temperature induced changes in the membrane composition may therefore play a role in the stability of such proteins leading to positive effects on whole plant growth at elevated temperature.

Studies on cyanobacteria have indicated that the mechanism by which temperature mediates compositional alterations in membranes is at the level of transcription. The level of desaturase transcripts in cyanobacterial cells increases in response to growth at low temperature [18]. It is likely that the transcription of cyanobacterial desaturase genes is dependent on the physical properties of the membranes themselves [19]. The idea that changes in the degree of fluidity of cyanobacterial thylakoid membranes might be a means of sensing thermal stress is supported by the finding that heat shock proteins were induced when the membrane fluidity was chemically altered [20]. However, most investigations conducted with plants suggest that transcription is not the primary means of controlling membrane fatty acid unsaturation. Studies examining the level of expression of desaturase genes in Arabidopsis have shown that the majority of them do not respond to either decreases or increases in temperature via changes in transcription [21-23], or to alterations in the membrane fatty acid composition due to mutations in the desaturase pathway [24,25]. Only the relatively unusual case of the desaturation step catalyzed by the Arabidopsis *fad8 *gene [25], and other highly specific examples for particular plant species [26], have been shown to be regulated at the level of transcription by temperature.

In this study we examined the leaf membrane fatty acid composition of Arabidopsis in response to temperature. After establishing the changes that occur at different elevated temperatures, we examined a number of mutant lines of Arabidopsis that possess well-characterized alterations in their membrane fatty acid content. The lines studied differ primarily in the degree of unsaturation of various lipids in chloroplast membranes. Examination of the fatty acid profiles of these lines grown at high temperature reveals a regulatory system that mediates membrane compositional changes even in the absence of specific desaturase steps. The results also suggest some general control points that might be important in the mechanism by which the membrane fatty acid unsaturation level is adjusted in response to temperature. In the course of carrying out these studies, we also examined the relationship between membrane fatty acid composition and growth at elevated temperatures.

## Results

### Elevated temperatures alter the membrane fatty acids of Arabidopsis

**Figure 1:**
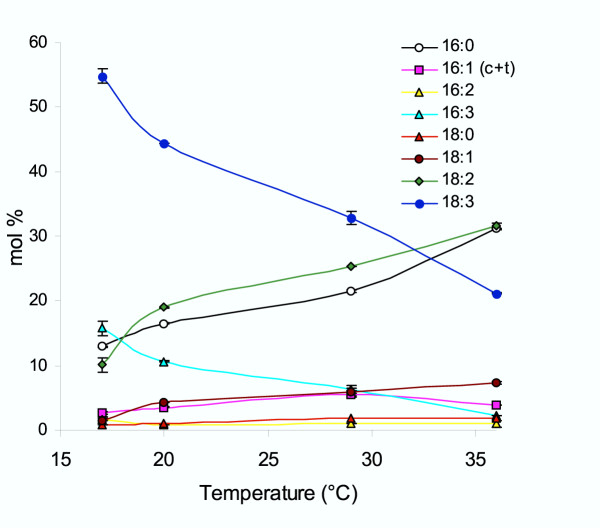
**Temperature modulates the leaf membrane fatty acid composition in Arabidopsis. **Wild-type Columbia plants were grown at various temperatures and total fatty acids were extracted from fully expanded rosette leaves. Line colors indicate the specific fatty acid as indicated in the legend. *c+t *indicates the combined amounts of *cis *and *trans *isomers of 16:1 fatty acids which co-elute under the gas chromatographic temperature profile used.

Arabidopsis lines were germinated and grown at 17°C for seven days before being shifted to designated temperatures. Total leaf fatty acids were extracted from lines grown at various temperatures for 30 to 35 d, converted to methylesters and analyzed by gas chromatography. Changes in the relative abundance of the major fatty acid species from the Columbia wild-type line grown at 17°C, 20°C, 29°C and 36°C were plotted and are shown in Fig. 1. The tendency of polyunsaturated species to decrease in abundance upon increasing growth temperature is evident within both 16 and 18 carbon-length fatty acids. The level of 18:3 in leaves decreased from 54 to 21 mol% of the total fatty acids from plants grown at 17°C and 36°C, respectively, and 16:3 species decreased from approximately 16 to 2.3 mol% for the same growth temperatures. In contrast, the proportion of diunsaturated linoleic acid (18:2) and monounsaturated oleic acid (18:1) showed an opposite response to elevated growth temperatures, with incremental increases of these species detected in plants grown at progressively higher temperatures. Specifically, the level of 18:2 increased from 10.1 mol% to 31.5 mol% of total fatty acids at 17°C and 36°C growth temperatures, respectively. These changes did not occur linearly over the temperatures measured but showed larger changes between 17°C and 20°C. At temperatures above 20°C, the relative abundance of the fatty acids generally changed in a more linear fashion. However, 16:0, unlike most other fatty acids, changed more abruptly between the two uppermost growth temperatures of 29°C and 36°C. To provide an additional estimation of the total membrane unsaturation level, the double bond index was calculated from the mol% fatty acid data (Table 2). The double bond index showed a corresponding decrease as the growth temperature was increased. Likewise, the levels of saturated palmitic acid, 16:0, accumulated to higher proportions in plants grown at elevated temperature, from 12.9 to 31 mol%. The relative abundance of other fatty acids (16:1, 16:2, and 18:0) showed alterations that were less pronounced in response to growth at elevated temperatures.

### Temporal evaluation of membrane alterations induced by high temperature

**Figure 2:**
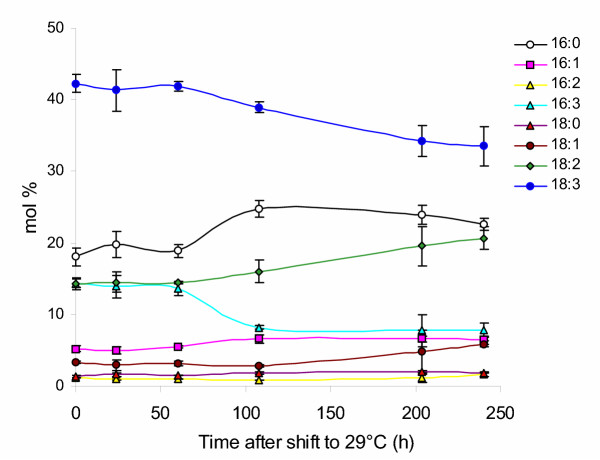
**Time-course analysis of the leaf membrane fatty acid composition in response to elevated growth temperature. **Plants were germinated and grown for 7 d at 22°C before being shifted to high temperature (29°C). Fatty acid profiles of wild-type Arabidopsis were determined by gas chromatographic analysis at the designated times over ten days after the shift to elevated temperature as described in materials and methods.

Based on the substantial changes detected after the shift to elevated temperature, we wished to determine whether these changes occurred on time scales similar to the timing known for new synthesis of fatty acids. To estimate the time required for a temperature increase to induce measurable changes in the membrane fatty acid composition, Arabidopsis seedlings were germinated and grown at 22°C for seven days and then shifted to 29°C. The fatty acid composition was then evaluated in rosette leaves sampled at various times after the shift to elevated temperature by gas chromatographic analysis (Fig. 2). The leaf fatty acid profile of plants grown at 22°C (0 h) was comparable to previous determinations made on plants grown at similar temperatures (Fig. 1). No significant changes were detected in the fatty acid profile 60 h after the shift to 29°C. The earliest time point reflecting an alteration in response to elevated temperature occurred at 108 h, with 16:0 and 16:3 exhibiting the largest and most abrupt shifts, and 18:2 and 18:3 showing detectable, but more gradual changes at this time. At the 204-h time point, 18:2 and 18:3 species exhibited alterations in their accumulation to a degree similar to those observed to change markedly in plants grown at different temperatures. Specifically, 18:2 increased in a relatively linear manner from approximately 16 to 19.5 mol% of the total while 18:3 decreased from about 39 to 34 mol% and these proportions remained relatively constant at the final time point measured, 240 h after the shift to high temperature.

The time course observed for the fatty acid composition to change in response to elevated temperatures correlates with the time determined for the incorporation of precursors to attain steady state levels in radiolabel feeding experiments [27]. The same general pattern of fatty acid changes was observed after the shift to high temperature. For example, 16-C fatty acids, 16:0 and 16:3, exhibited an abrupt shift from 18.9 to 24.8 mol% and 13.6 to 8.2 mol%, respectively, at the 108-hr time point, and remained relatively constant, until the final time point, except that 16:0 tended to decrease somewhat, after the increase observed at 108 h. On the other hand, major changes in the relative proportions of 18-C fatty acids, a large percentage of which are derived through the eukaryotic pathway, were not evident until the 204-h time point. This distinction between the onset of changes of 18:2 and 18:3 and of 16:0 and 16:3 is also reflected in the more linear response of 18:2 and 18:3 compared to the more abrupt transition and then fairly linear response of 16:0 and 16:3. Control experiments in which wild-type Arabidopsis lines were maintained at 22°C and sampled for membrane fatty acids over the same time intervals as shown in Figure 2, did not show equivalent alterations at 108 h but maintained similar profiles throughout (data not shown). Thus, detection of the first alterations in the fatty acid composition in response to increased growth temperature is consistent with the time required for new synthesis of fatty acids. These results also suggest that changes in membrane fatty acids in response to temperature require turnover and resynthesis of new lipid to achieve the temperature-adapted composition.

### Temperature-induced changes in membrane fatty acid composition of mutant lines to probe membrane regulation

A number of Arabidopsi*s *lines possessing well-characterized alterations in their membrane fatty acid profiles (Table 1) were used to examine how a specific block in the glycerolipid pathway affects the membrane composition in response to high temperature. For this analysis, we examined the membrane fatty acid content of plants grown at 17°C and 36°C. As in the previous determinations (Fig. 1), all plants were germinated and grown in an environmental chamber at 17°C for seven days before shifting to one set to 36°C. The fatty acid profile was then determined after 25–30 d of growth. The lower growth temperature of 17°C was chosen based on the response of the membrane fatty acid composition of leaves to this temperature determined previously, as well as the overall growth performance of the plants. The upper temperature of 36°C was selected based on the pronounced alterations observed in the fatty acid composition of wild-type plants grown at this temperature and the fact that this temperature represents the physiological upper limit for vegetative growth in soil. Indeed, growth at this temperature led to sterile plants (data not shown). This loss of seed production was apparently due to insufficient pollen formation and was consistent with notable decreases in seed yield (approximately 50%) from lines grown at 29°C (data not shown). Incubation at 36°C was also used to provide conditions by which the leaf fatty acid profiles would be most clearly delineated among the various Arabidopsis mutant lines as well as to establish the magnitude of the alterations in the fatty acid composition in response to elevated temperature.

**Figure 3:**
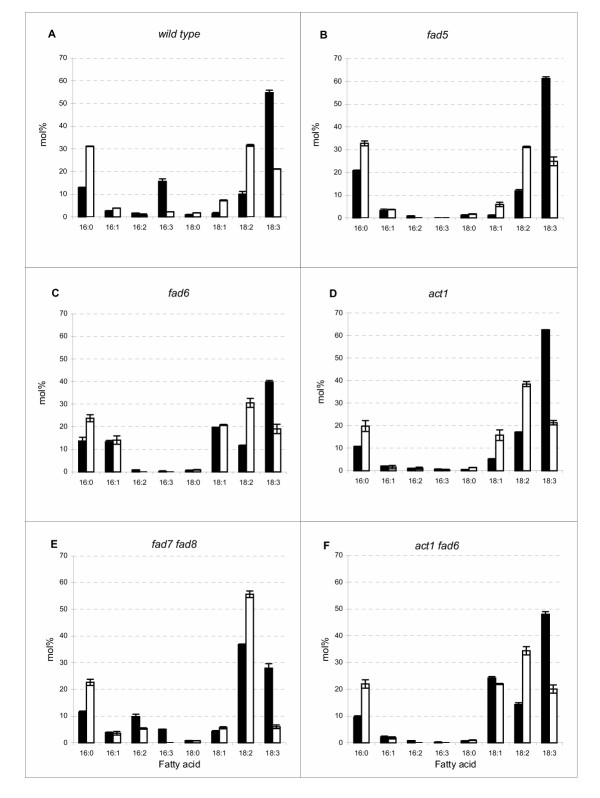
**Leaf fatty acid profiles obtained from Arabidopsis lines grown at 17°C (black bars) and 36°C (white bars). **(A), wild-type Columbia. Mutant lines: (B) *fad5*; (C) *fad6*; (D) *act1*; (E) *fad7 fad8*; (F) *act1 fad6*.

Each line used in this analysis possessed a mutation that primarily impacts the composition of the photosynthetic membranes of the chloroplasts (Table 1). Several of these mutants have been shown previously to display variable degrees of thermal tolerance [28-31]. Examination of the fatty acid profiles from these lines reflect the same general trends seen for wild-type plants grown at temperatures above 17°C (Fig. 1). As observed previously, the level of polyunsaturated fatty acids generally decreased with a concomitant increase in diunsaturated and saturated fatty acids when compared to plants grown at 17°C (Fig. 3). Specifically, trienoic fatty acids (16:3 and 18:3) decreased sharply and the level of dienoic 18:2 (but not 16:2) increased at the elevated temperature. Saturated 16:0 and, in some lines, monounsaturated 18:1, also accumulated to higher levels in plants grown at 36°C. A general estimation of the membrane unsaturation level was also provided by the double bond indices derived from this data (Table 2). Mutant lines *fad5*, *fad6*, *fad7 fad8*, and *act1 fad6 *showed a lower double bond index from plants grown at 17°C compared to wild type, with *fad6 *displaying the most pronounced decrease in overall unsaturation. In contrast, the double bond indices obtained from membranes of the mutant lines grown at 36°C were similar to each other and the wild type with an average value of 1.5. Thus the total membrane unsaturation level is relatively equivalent among the different lines grown at the higher temperature.

Two principal changes were apparent among the individual lines in response to high temperature: an increase in the abundance of 16:0 and 18:2 fatty acids and a pronounced decrease of both trienoic polyunsaturated species, 16:3 and 18:3 (Fig. 3). In the case of the *fad5 *mutant line, which is deficient in the desaturation of 16:0 to 16:1 at the *sn*-2 position only on chloroplastic monogalactosyldiacylglycerol [32], the 16-C fatty acids from plants grown at 17°C resembles those of the wild type grown at high temperature (Fig. 3B). This includes the proportion of 16:3, which is essentially absent due to the block in the prokaryotic pathway at the level of 16:0. At 36°C, the composition of *fad5 *membranes is most similar among all of the mutants examined to that of the wild type grown at high temperature. The high degree of similarity of the *fad5 *and wild-type compositions may implicate the step catalyzed by the FAD5 desaturase to be an important control point in the acclimatization of the membrane composition in response to temperature.

Examination of the membrane fatty acid composition of the *fad6 *mutant line in response to high temperature similarly reveals the general trends seen in the wild-type line grown at elevated temperature (Fig. 3C). Because *fad6 *is deficient in the desaturation of 16:1 and 18:1 to 16:2 and 18:2, respectively, on all chloroplastic lipids [33], the accumulation of both 16:1 and 18:1 in this line are evident. The proportions of these two monounsaturated fatty acids remain virtually unchanged in *fad6 *plants grown at low and high temperature, showing that the overaccumulated levels of these species, due to the mutation in the FAD6 desaturase, is not subject to alterations in response to temperature.

The mutation in the *act1 *line blocks entry of fatty acids into the prokaryotic pathway by a deficiency in chloroplast GPAT [34]. This is reflected in the fatty acid composition of *act1 *plants grown at both low and high temperature, with a decrease in all 16-C fatty acid species (Fig. 3D). Temperature-induced changes in the fatty acid composition of the *act1 *line shows a profile highly similar overall in 18-C fatty acids compared to the wild type, suggesting that the desaturases controlling the conversion of 18:2 to 18:3 is a major control point capable of adjusting unsaturation levels of leaf membranes. The slightly elevated level of 18:2 at both temperatures in *act1 *compared to the wild type likely reflects the previously demonstrated increase of fatty acid flux into the chloroplast membranes via transfer of primarily this fatty acid [27,34].

The deficiency in the *fad7 fad8 *line is due to mutations at two loci, each encoding desaturase isozymes that convert 16:2 and 18:2 to 16:3 and 18:3, respectively, in the chloroplast [35]. In this line, the dienoic fatty acids, 16:2 and 18:2, are elevated and the effect of high temperature resulted in a reduction of this elevated 16:2 from 9.8 to 5.4 mol% (Fig. 3E). The proportion of 18:2, on the other hand, increased in response to high temperature from 36.8 to 55.7 mol%. The trienoic fatty acid, 18:3, which due to the *fad7 fad8 *mutations was lower than the level in the wild type at 17°C (28 vs 54.8 mol% in the wild type), decreased to 6 mol% of total fatty acids at 36°C, the lowest leaf 18:3 levels of all the lines tested. Thus, the general trend of increasing 18:2 and decreasing 18:3 in response to high temperature growth was retained in the absence of most FAD7 and FAD8 desaturase activities, which catalyze the formation of the majority of trienoic fatty acids in the leaf membranes.

A line derived from a cross between *act1 *and *fad6 *was produced during this study and used to evaluate the performance and temperature response of plants containing a membrane fatty acid composition which is distinguished by an elevated level of 18:1 (due to the deficiency in the FAD6 desaturase) but no increase in the relative amounts of 16:1 (because of the block into the prokaryotic pathway, due to diminished GPAT activity). The resulting composition in the *act1 fad6 *double mutant line grown at 17°C indicates the profile expected for mutations at each of these steps. The fatty acid profile altered in response to elevated temperature is similar to each parental mutant line grown at high temperature (Fig. 3F). The *act1 fad6 *line thus provides a membrane fatty acid profile dissimilar to other lines in this study in that it contains elevated 18:1 but no increases in the proportion of 16:1 or 16:3. Such a profile can be used to address the functional significance of having only increased 18:1 but relatively similar proportions of all other fatty acids (see below).

### Fluorescence yield enhancement

Six Arabidopsis lines possessing distinct membrane fatty acid compositions were evaluated by measuring chlorophyll fluorescence parameters to determine the thermal stability of PSII. The use of chlorophyll fluorescence has been used extensively to investigate photosynthesis and previous studies have examined photosynthetic stability using similar methods on isolated chloroplasts or detached leaves from the *fad5 *and *act1 *lines [29,36]. However, the analysis conducted here is the first to use *in planta *measurements in side-by-side comparisons to examine differences in thermal tolerance. In addition, prior to the fluorescence stability measurements, the fatty acid compositions of all lines used in this analysis were determined and all were found to be within the standard error of those values determined for the fatty acid profiles of the lines grown at 17°C (Fig. 3, data not shown). Table 3 shows the results of the fluorescence analysis. Plants were grown at 17°C and evaluated at approximately four weeks of age. In all plants grown at this temperature, it is apparent that mutant lines *fad6*, *fad5 *and ac*t1 *show a statistically significant increase in the fluorescence transition point (TP) temperature while *fad7 fad8 *shows a slight but insignificant increase. These results are likely to be due to differences stemming predominantly from the distinct membrane fatty acid compositions in leaves from the lines grown at this temperature. The TP values obtained for these intact leaf measurements here, are very similar to various determinations previously conducted on some of the lines using isolated chloroplast preparations or detached whole leaves [29,36,37]. Thus, only some lines display enhanced stability of photosynthetic quantum yield as the leaf temperature is increased and this seems to be positively correlated with a decrease in trienoic fatty acids, particularly 16:3, derived from the prokaryotic pathway. However, the relative proportion of 16:3 does not appear to be the only determinant for increased thermal stability.

The double mutant *act1 fad6 *provided a line with a fatty acid profile that is distinct from that of other lines analyzed in this study (i.e., an increase in the percentage of 18:1 but no increased level of 16:1 at 17°C) (Fig. 3F). To determine if this composition resulted in differences in the photosynthetic thermostability, we subjected the *act1 fad6 *line to fluorescence measurements. The *act1 fad6 *double mutant did not show a significant difference in the TP temperature from that of the wild type, even though each of the parental lines that possess only the single respective mutation exhibited a measurable enhancement of thermal stability (Table 3).

The *act1*^++ ^line is a transgenic line that over expresses GPAT (i.e., the *act1 *gene product). It was included as an additional line here to test whether its fatty acid composition would impact the thermal stability measurements as determined by chlorophyll fluorescence. However, the leaf fatty acid composition of *act1*^++ ^plants grown at 17°C is similar to the wild-type composition except that the percentage of 16:0 and 16:3 fatty acids is increased to 2.0 and 1.5 mol%, respectively, over the proportions determined in the wild type. No differences in thermostability as indicated by chlorophyll fluorescence measurements were apparent for this line compared to the wild type.

Although the most equivalent comparisons for stability measurements among the different lines would be obtained from those grown at temperatures considered moderate for Arabidopsis, such as 17°C used here, we also determined the TP temperatures for those lines that exhibited enhanced thermal stability at 17°C after growth at high temperature. Since this study revealed clear differences in the membrane fatty acid composition at various elevated temperatures, an indication of high-temperature acclimation based on fluorescence measurements may be evident. Four plants including wild type were grown at 29°C for three weeks before conducting the fluorescence stability tests. The results indicated an increase in the TP temperature from 42.7°C for wild type grown at 17°C to about 44.8°C in plants grown at 29°C (Table 3). However, the *fad5*, *fad6 *and *act1 *lines, which showed reproducible increases in thermal stability compared to wild type when grown at 17°C, showed essentially the same TP temperatures as the wild type when grown at the elevated temperature. The lack of a detectable increase in thermal stability in mutant plants above the temperature found for the wild-type line grown at high temperature may be a consequence of the altered composition of the membrane fatty acids after growth at high temperature. The similar TP temperatures are also consistent with the overall similar unsaturation levels as indicated by the double bond index calculated for these lines. These results may also reflect the upper tolerance limit attainable that arises from fatty acid adjustments in response to elevated growth temperature, at least with the different compositions in the lines studied here.

### Growth at elevated temperature

Most of the mutant lines used in this study were chosen on the basis of their altered fatty acid profiles in chloroplast membranes. As shown here, reductions in the total level of triunsaturated fatty acids in many lines grown at 17°C reflects the composition of the wild-type line grown at elevated temperature. To establish whether the observed changes translates into improved performance at the whole plant level, growth rates were determined for the lines at various temperatures. Growth rates were determined only during the first six days after the shift to elevated temperatures in order to maintain equivalent membrane fatty acid compositions of the lines before changes occurred in membranes during acclimation to high temperature (Fig. 2). As shown in Table 4, the relative growth rates of *fad5*, *fad6*, *act1 *and *fad7 fad8 *seedlings are at least the same or slightly higher than wild type within several days after the shift to high temperature. In agreement with findings by Murakami et al. [31], the *fad7 fad8 *mutant line exhibited the most pronounced tolerance to high temperature based on growth rate. These results indicate that a less polyunsaturated membrane fatty acid composition than normally found in wild type favors seedling growth at elevated temperatures.

## Discussion

Several clear alterations are evident in the membrane fatty acid composition in response to high temperature growth. A decrease in trienoic fatty acids, including strongly diminished 16:3, produced exclusively within the prokaryotic pathway in the chloroplast, is consistent with the general decrease of polyunsaturated fatty acids in response to high temperature. Concurrent with the reduced accumulation of trienoic fatty acids at high temperature is an increase in linoleic acid, 18:2, and an increase in 16:0 (Figs. 1 and 3). The observation that 16-C fatty acids show a pattern distinct from that of 18-C fatty acids suggests that individual fatty acid classes may have specific roles in maintaining optimal membrane function as well as different mechanisms governing their synthesis. This idea of distinct roles for particular fatty acids in the membrane is also supported by the relatively similar degree of membrane unsaturation in most of the lines examined (Table 2), despite the differences found in growth or photosynthetic stability.

It is remarkable that these general alterations are observed even in mutant lines that are deficient in steps early or late in the desaturation pathways. For example, the profile of *fad5*, which is deficient in 16:0 desaturation in the chloroplast compared to *fad7 fad8*, which is deficient in the final desaturase step forming trienoic fatty acids, show similar overall trends in response to high temperature. These temperature responses must take into account the two glycerolipid pathways that operate in parallel in Arabidopsis, one in the chloroplastic envelope membranes and one in the endoplasmic reticulum [38,39].

Previous characterizations of all of the individual mutant background lines used in this study have clearly indicated the primary lipid species acted upon by the specific desaturase (or acyltransferase) activity missing in each mutant (Table 1). The temperature-induced alterations in the membrane composition can be considered in the context of the particular mutant background with its corresponding deficiency in a given step of the glycerolipid pathway. In this case, the mutations examined primarily affect chloroplastic lipids. In addition, the polyunsaturated 16-C fatty acids can be used as reliable markers for the major chloroplastic lipids monogalactosyldiacylglycerol and digalactosyldiacylglycerol, since they are the only lipids that contain these fatty acids. In regard to lipid compositions following growth at elevated temperature, examination of the proportions of individual lipids from the related species, *Brassica napus*, has shown that significant changes do not occur in leaf membranes from plants grown at 20°C and 30°C [15]. This finding suggests that it is the degree of fatty acid unsaturation that varies most appreciably at these temperatures and not the levels of the major leaf lipids themselves [15].

### Temporal basis for temperature-induced membrane alterations

The time required for the fatty acid composition to adjust to high-temperature growth conditions demonstrated that major alterations in leaf membranes do not occur rapidly in response to elevated temperature. The ~60-h period before changes become evident in the fatty acid profile corresponds with the occurrence of new lipid synthesis and turnover and therefore does not suggest a mechanism by which temperature induces modifications of existing membrane fatty acids. These results are also consistent with labeling studies that show fatty acids produced by the prokaryotic pathway accumulate prior to those synthesized via the eukaryotic pathway. The abrupt changes in 16-C fatty acids beginning 60 h after the shift to high temperature compared to the gradual and more continuous alterations observed for 18-C fatty acids (Fig. 2) also fits with these labeling patterns. It is well established by a number of time-course radiotracer labeling studies, including several conducted on most of the mutant lines used here, that earlier stages in the glycerolipid pathway show alterations prior to those formed through the eukaryotic pathway [27,33-35,40]. These include the formation of saturated fatty acids prior to the accumulation of unsaturated species and the earlier production of palmitate-containing species in the prokaryotic pathway. Overall, these time-course observations suggest that the modulation of membrane unsaturation levels plays a role in longer-term acclimation of the plant. Transient fluxes in environmental temperature are therefore not likely to result in pronounced alterations in the composition of leaf membranes.

### Membrane composition in response to temperature in mutant lines

The fatty acid composition resulting from high-temperature growth in the different genetic backgrounds reveals a complex regulatory system. Thus, despite deficiencies in several enzymatic steps in the different mutants, the membrane fatty acid composition undergoes adjustments similar to those observed in the wild type in response to elevated temperature. The similar increase in 18:2 levels after high temperature growth in the *fad5*, *fad6*, *act1*, *act1 fad6 *and wild-type lines suggests that the percentage of 18:2 may be important in membrane acclimatization. These results also suggest that desaturase activities as well as flux from extrachloroplastic membranes might also be controlled to mediate the response to temperature (see below). The accumulation of 18:2 (as opposed to 18:1) in the lines tested implicates it as a preferred species in membranes adapted to high-temperature growth. Although unknown, one speculation for 18:2 for this may be due to the "intermediate" level of disorder represented by diunsaturated acyl chains in the membrane. Fatty acids with one double bond, such as 18:1, impart a greater relative degree of disorder to the membrane than acyl groups containing two double bonds which, in turn, confer only slightly less disorder to the membrane relative to triunsaturated 18:3. The composition of the *fad7 fad8 *mutant line supports this contention, in which the mol% of 18:2 is almost 2-fold higher than that of wild-type membranes and this line exhibited the best growth at elevated temperature. However, biological membranes are complex, dynamic structures and it is likely that other, unknown factors will be important to maintain optimally functioning membranes.

The fatty acid profiles of several mutants grown at high temperature suggests that the control of flux of fatty acids from the eukaryotic pathway is partly responsible for the changes observed in 18:2 and 18:3 due to temperature. In the *fad6 *mutant grown at high temperature, the proportions of 18:2 and 18:3 are highly similar to the proportions observed in the wild type, despite that essentially all of the 18:2 must be derived from the eukaryotic pathway. Labeling studies have shown previously that the *fad6 *line exhibits a decrease in lipid synthesis via the prokaryotic pathway [33], and this same mechanism, which presumably operates to maintain specific physical properties of the chloroplast membranes, may also be involved in mediating compositional adjustments of the membranes in response to increased temperature. In this case, a possible mechanism might be a reduction in the amount of 18:2 fatty acids transferred to the chloroplast for desaturation by the FAD7 and FAD8 desaturases. The *fad6 *profile also reveals that activity of the FAD2 enzyme, operating in the endoplasmic reticulum, might also be subject to temperature regulation.

Similarly, in the *act1 *mutant line, in which entry of fatty acids into the prokaryotic pathway is blocked by the step catalyzed by GPAT, the primary difference is a decrease in the level of 16:0 when grown at elevated temperatures. This presumably reflects an increase into the eukaryotic pathway but with a similar temperature-responsive regulation. Flux of 18:2 from the eukaryotic pathway back into the chloroplast also appears to be modulated, as the proportions of 18:1 and 18:2 are slightly elevated in *act1 *at both temperatures, while the 18:3 level is highly similar to wild type.

The fatty acid composition of the *fad7 fad8 *mutant reveals alterations that occur in response to elevated temperature in the absence of all trienoic fatty acid-forming desaturase activity in the chloroplast (Fig. 3E). This profile also suggests that transfer of fatty acids from the eukaryotic pathway may be an important component in temperature regulation of the membrane composition. Such a mechanism is possible considering that the major proportion of 18:3 produced in this line must be synthesized through the eukaryotic pathway via the FAD3 desaturase [27]. The resulting low level of 18:3 detected in plants grown at high temperature is evidence that the FAD3 enzyme in the endoplasmic reticulum also is subject to temperature regulation. Thus it appears that desaturase enzyme activity is inversely regulated by increased temperature, in agreement with previous proposals as a likely mechanism [41]. Analysis of the data presented here suggests that the desaturases that catalyze trienoic fatty acid formation (FAD7, FAD8 and FAD3) and the FAD5 desaturase are the enzymes likely to have the greatest impact if regulated in this way.

### Membrane fatty acid composition has a role in enhancing photosynthesis to tolerate high temperature

Studies conducted on the ability of plants to acclimate to elevated temperature have mainly focused on components most likely to affect the stability of photosynthetic electron transport, particularly PSII. In this respect, the composition of the chloroplast thylakoids is expected to be important in the thermal tolerance of photosynthetic electron transport [10]. Moon et al., [42] have implicated the unsaturation level of PG as being important in the removal and replacement of damaged D1 proteins in plants. However, the direct relationship pointing to protein-lipid associations being involved in stabilizing the D1 protein at high temperature has only recently been suggested [43]. The results presented here imply a regulatory mechanism that confers a similar overall composition in response to temperature regardless of the initial fatty acid alteration in the membranes due to mutation. A possible reason for such apparently stringent control might be that compositions that are deleterious for membrane function are curtailed, utilizing desaturase pathways that are present in both chloroplastic and extrachloroplastic locations.

Measurement of PS II activity during leaf heating was used here as a sensitive indicator of the thermostability that might be conferred by the different membrane compositions. In this case, the temperature at which the quantum yield of PS II electron transport collapses (TP) was determined in intact leaves. The fluorescence measurements were conducted on plants grown at 17°C, to minimize variation and other potential responses, such as increases in the synthesis of heat shock proteins that might be induced at higher temperatures. In addition, only at the 17°C growth temperature were differences observed in the total membrane unsaturation level among the different lines tested as estimated by the double bond index, which might suggest that the largest influence of the fatty acid composition occurs at lower and more moderate temperatures.

The *fad6 *mutant exhibited the maximum fluorescence yield enhancement of the lines grown at 17°C. This maximum TP is essentially the same as that obtained from several plants grown at 29°C, including the wild-type line (Table 3B). The 2°C difference apparent in the mutant lines grown at 17°C may be an accurate indication of the magnitude of PSII thermal stability that can be conferred by adjustments in the membrane fatty acid composition and therefore may reflect the extent to which these adjustments can contribute to high-temperature acclimation in Arabidopsis. While it is unknown how specific fatty acids influence thermal stability, analysis of the *act1 fad6 *mutant described in this study demonstrates that alterations in the relative abundance of 16:1 and 18:1 have an effect. Both *act1 *and *fad6 *mutant plants display enhanced stability compared to wild type as determined by fluorescence measurements whereas the *act1 fad6 *line, which does not exhibit elevated 16:1 but slightly higher levels of 18:1, shows no statistical difference in the TP temperature compared to wild type (Table 3). This difference in fluorescence TP temperatures among these three mutants, as well as a lack of a correlation between the TP values and the double bond index, illustrates that the relative level of a specific lipid class can influence thermal stability. Measurements of diffusion rates of light-harvesting complexes in desaturase mutants of cyanobacteria also point to distinct roles that lipids may have in photosystem stability. In a recent study, the interaction of phycobilisomes with reaction centers was proposed to be stabilized by lipids as opposed to being affected by the level of membrane unsaturation directly [44]. Studies using spectroscopic methods have also suggested that overall lipid acyl chain disorder in cyanobacterial membranes is similar despite differences in growth temperature or unsaturation levels [45] and that protein-to-lipid interactions in membranes seem to be a key parameter in membrane dynamics [45,46].

Measurements of a single physical parameter attributable to alterations in the membrane composition have often not correlated with results apparent in whole plant performance tests. Similar to the results presented here, Murakami, et al., [31] have shown that chlorophyll fluorescence measurements might not be the ideal indicator to assess whole-plant performance. For example, although the *fad7 fad8 *line showed the fastest growth rate at high temperature, it did not show a significant difference in thermal stability based on fluorescence yield. The use of other functional measurements, such as CO_2 _uptake rates or O_2 _evolution, may provide indicators that more reliably assess potential high-temperature tolerance, although these measurements can also lead to unpredictable results regarding whole-plant physiology. In an Arabidopsis mutant devoid of virtually all digalactosyldiacylglycerol in chloroplast membranes, O_2 _evolution was found to be unaffected despite large changes in thylakoid membrane organization and in fluorescence energy transfer characteristics [47]. Thus it is likely that additional differences stemming from the relative levels of distinct fatty acids in the membrane affect thermal tolerance and that membrane unsaturation levels will not be the exclusive factor. Other induced changes that impact membrane structure and function can be important. For example, alterations in the length and positions of double bonds within the acyl chain of a lipid molecule can confer widely differing properties and suggest that specific proportions of distinct fatty acid classes may be necessary for optimum membrane function [48].

A number of processes are called into play in response to stresses such as high temperature. Induction of heat shock proteins serves as one countermeasure to respond to elevated temperatures [5] and their specific roles in thermal tolerance are becoming clearer [2-4,49,50]. Correlations between the antioxidant status of plants and thermotolerance have also been noted recently [51-53]. Additional, perhaps more species-specific, responses are likely to be important in protecting against harmful effects of high temperature growth, including the accumulation of small molecules such as glycinebetaine and through the regulation of carbon fixation via rubisco activase [6,54].

Although decreases in the amounts of trienoic fatty acids may be an important determinant for plant thermal tolerance, lines possessing elevations in other, distinct fatty acid species, exhibit different characteristics. A triple desaturase mutant of Arabidopsis demonstrated that all trienoic fatty acids in leaf membranes are not essential for growth at low temperature [55]. This *fad7 fad8 fad3 *mutant also displayed thermostability but actually died after prolonged exposure to 33°C, suggesting that some trienoic fatty acids are indeed essential for high temperature growth [56]. The upper limit of chronic high temperature exposure for all lines tested here was about 34°C, where plants continue to grow and flower but exhibit reduced seed yield (data not shown), consistent with the idea that some trienoic fatty acids are essential. Although not addressed in the present study, it would be of interest to determine if this decreased seed yield from high-temperature-grown plants was related to insufficient levels of linolenic acid, a precursor required for the synthesis of jasmonic acid, a signaling molecule necessary for pollen development [57].

The results relating to growth performance at high temperature in this study are noteworthy in view of the fact that Arabidopsis is not considered a high temperature tolerant plant [58]. In more heat tolerant species, adjustment of the membrane fatty acid composition may well have greater significance in providing a rational means to control plant high-temperature tolerance. For example, suppression of a FAD7 homolog to concomitantly raise 18:2 and decrease 18:3 in tobacco enabled enhanced growth at elevated temperature [31]. Thus elimination of trienoic fatty acids might be the most critical aspect of altering the membrane composition to favor such enhanced growth. It is unclear, however, if the corresponding increases in monounsaturated and diunsaturated fatty acids that occur in such lines also contribute to the improved tolerance. Several of the mutant lines examined in this study also possessed very low levels of trienoic fatty acids but were accompanied by distinct alterations in other fatty acids. Most of the mutant lines exhibited elevations in the fatty acid species that serves as a precursor to the mutated step. The *fad5 *line grown at 17°C displayed 16-C fatty acids that were most similar to that of the wild-type line grown at 36°C, and had a membrane fatty acid profile almost identical to the wild type when each was grown at 36°C (Fig. 3A,3B). While such a composition has no deleterious effects at moderate or elevated temperatures, this line, as well as the *fad6 *line, shows impaired growth and chloroplast development at low temperature [28]. Based on the similar composition to high temperature-grown wild-type plants, the reaction catalyzed by the FAD5 desaturase, which has been identified as an expressed sequence tag [59], may be an additional target to further manipulate tolerance to high temperature.

## Conclusions

This study has shown that temperature precisely modulates the membrane fatty acid composition and these changes occur via mechanisms that are not profoundly affected by a number of mutations in the fatty acid desaturation pathway. These observations support the idea that the unsaturation level of plant membranes plays some role in enabling the plant to tolerate elevated temperatures but other characteristics of membrane lipids will likely also be important. Examination of a number of fatty acid desaturase mutants also suggests that alterations in the unsaturation level of membrane fatty acids in response to growth temperature apparently occurs by controlling the level of desaturase activity at several steps within the lipid biosynthetic pathway as well as by modulating inter-compartmental flux between the chloroplastic and cytosolic pathways.

## Methods

### Plant material and growth conditions

Arabidopsis lines were grown in environmentally controlled chambers (Conviron, Inc.) adjusted to a given temperature under continuous light at 120–150 μmol m^-2 ^s^-1^. Seeds were sown on pots containing vermiculite-perlite mix (1:1:1) potting soil irrigated with a commercial fertilizer at every third watering. For growth in pots at elevated temperatures, seeds were germinated and grown at 17°C for seven days and then shifted to growth chambers adjusted to the respective temperatures and set to maintain humidity levels to at least 80% or higher, which was found to be essential to maintain growth at temperatures above 32°C. These chamber-grown plants were utilized for determining leaf membrane fatty acid compositions and for analyzing fluorescence yield characteristics. Growth rates of seedlings were determined on lines germinated and grown in Murashige and Skoog salts media (Sigma) containing 1% sucrose and 0.7% agar. Surface-sterilized seeds were plated, treated at 4°C for 2 to 3 days to synchronize germination, and grown for 16 d at 16°C in a growth chamber before shifting to elevated temperatures. Light intensity remained constant at 60 μmol m^-2 ^s^-1^. Four seedlings were sampled at days 0, 2, 4 and 6 d after the shift to elevated temperatures and were dried for 2 d at 70°C before weighing. The relative growth rates (ω^-1^) were calculated as the slope of the natural log of the seedling dry weight plotted over time in days. Growth rate determinations for seedlings grown at 22.5°C and 30°C were conducted 3 separate times to obtain SE and growth rates determined for 28 and 34°C were conducted once.

The Arabidopsis mutant lines used in this study are listed in Table 1. All of these are descendents of the Columbia ecotype. The *act1 fad6 *double mutant line was derived by first crossing *act1 *to the *fad6 *line and then crossing progeny from the resulting heterozygotes back to *fad6 *before selecting for lines possessing a fatty acid composition indicative of both homozygous mutations. The *act1*^++ ^line, is a transgenic line containing the gene encoding glycerol-3-phosphate acyltransferase (GPAT) (Schneider and Somerville, unpublished, [60]) under the control of a dual 35S cauliflower mosaic virus promoter. It was included as an additional control for the chlorophyll fluorescence studies and possesses a membrane fatty acid composition virtually identical to the wild-type profile, except for a very slight elevation in the amount of 16:3 (~1.5 mol%, over the percentage in wild type at 17°C).

### Fatty acid analysis

For each determination, at least two plants were used as a source of leaf material for extraction and analysis. Three mid-to-fully expanded (2 to 5-cm long) rosette leaves were harvested from each plant by removing approximately one-third of the leaf and immediately storing at -80°C in Teflon-capped tubes until needed. Preparation of methyl esters from these leaves and gas chromatographic analysis of the resulting extracts was performed using established procedures [61] with a Hewlett-Packard 5800 series gas chromatograph equipped with Supelco SP2330 glass capillary column (0.75-mm × 20-m). An estimation of the membrane total unsaturation level (double-bond index) was calculated from the mol% values derived from the gas chromatographic data, according to the equation: [Σ(mol% fatty acid content × no. of double bonds)]/100 as described by Skoczowski, et al. [62].

### Fluorescence measurements

Chlorophyll fluorescence was measured using an Opti-Sciences OS500-FL pulse modulated fluorometer (Opti-Sciences, Inc. Tyingsboro, MA). A custom designed aluminum heating block was constructed to hold an Arabidopsis rosette leaf, the associated optical cables and the temperature probes to conduct the leaf chlorophyll excitation and fluorescence measurements. An Arabidopsis leaf (~2.4 cm long) was fitted into a recessed (~0.5-mm) portion of the heating block to prevent damage to the leaf section. An ethylene glycol solution was circulated within the block to control temperature. The block and clamp module also provided an adjustable fixture to stably hold the leaf of a potted plant during the measurements.

The photosynthetic thermostability of the different lines was evaluated by determining the transition point temperature of fluorescence quantum yield (TP). An attached rosette leaf was fitted into the thermally controlled heating block and fluorescence was monitored with a saturating light pulse (0.8 s duration) every 90 s. After stabilization at 29°C (4–5 pulses), the temperature of the heating block was increased at a rate of 0.75°C min^-1 ^to a final temperature of 48.5°C using a digital temperature control unit (Omega Technologies Co. Stamford, CT). Leaf temperatures indicated were measured with a thermocouple in contact with the leaf surface. Quantum yield was calculated by the equation F'_ms_-F_s_/F'_ms _according to Genty [63], where F_s _is the steady state fluorescence under environmental conditions, and F'_ms _is the maximal fluorescence yield obtained after a saturating light pulse. F_m_-F_o_/F_m_, which is a measure of the photochemical efficiency of photosystem II, is based on the variable fluorescence (F_m_-F_o_), which is the minimum (F_o_) and maximum (F_m_) fluorescence of a dark adapted leaf before and after, respectively, of a saturating light pulse. All determinations were made on leaf samples that gave an initial F_m_-F_o_/F_m _ratio of at least 0.80, prior to initiating the temperature increase. This measure was taken to ensure that the integrity of the leaf section introduced into the clamp module was sound and to verify that the leaf was properly fitted into the measurement module. The mean value ratio of 0.83 is indicative of a well functioning photosynthetic apparatus in an unstressed leaf as has been shown in a variety of other plant species [64].

## Authors' contributions

DF executed the fatty acid analysis, photosynthetic measurements, data analysis and drafted the manuscript. JO undertook the production of the *act1 *overexpressing lines and assisted in the construction of the double mutant line. CS conceived of the study. All authors read and approved the final manuscript.

## Figures and Tables

**Table 1:** Arabidopsis lines used in this study. All lines contain mutations that primarily affect the composition of chloroplast membranes involved in photosynthesis.

Line	Biochemical defect	Primary affect on membrane composition	Reference
*fad5*	16:0 to 16:1 desaturation on *sn*-2 of all chloroplast galactolipids	Elevated 16:0; no 16:3 in chloroplast membranes	[32]
*fad6*	16:1 to 16:2 and 18:1 to 18:2 desaturation on *sn*-1 and *sn*-2 of all chloroplast lipids	Elevated 16:1 and 18:1; no 16:3, diminished 18:2, 18:3	[33]
*fad7 fad8*	16:2 to 16:3 and 18:2 to 18:3 desaturation on *sn*-1 and *sn*-2 of all chloroplast lipids	Elevated 16:2 and 18:2; diminished 16:3 and 18:3	[35]
*act1*	Plastid glycerol-3-phosphate acyltransferase (GPAT) activity; first step in the prokaryotic pathway	Slightly elevated 18:1; diminished levels of 16-C fatty acids (prokaryotic pathway)	[34]
*act1 fad6*	Double mutant containing both the *act1 *and *fad6 *mutations	Slightly elevated levels of 18:1 (than *act1 *alone); no elevation of 16:1 (as in *fad6*); prokaryotic pathway blocked	This work
*act1*^++^	Plastid GPAT overexpressor	Similar to wild type. Slightly elevated levels of 16:3, reflecting slightly enhanced flux through prokaryotic pathway	This work

**Table 2:** Double bond indices of lines grown at 17°C and 36°C. The double bond index was calculated for each line from the mol % fatty acid values indicated in Figure 3.

Line	Growth temperature	
	17°C	36°C
WT	2.39	1.46
*act1*	2.33	1.62
*fad5*	2.13	1.46
*fad6*	1.78	1.53
*fad7 fad8*	2.00	1.49
*act1 fad6*	2.01	1.53

**Table 3:** Temperature (TP, °C) at which fluorescence yield enhancement occurs in several Arabidopsis lines. Plants were grown at 17°C or 29°C and fluorescence was analyzed continuously using an OSI systems fluorometer during heating at 0.75 °C min^-1^, as described in methods. TP is the transition point temperature corresponding to 50% of the maximal quantum yield due to leaf heating. Values are the means ± SE on six determinations conducted on two different plants for 17°C-grown plants and four determinations for 29°C-grown plants. Asterisks indicate significant differences from the value obtained for wild-type plants, * P < 0.05; ** P < 0.01. ND, not determined.

	17°C-grown		29°C-grown
Line	TP (°C)	°C above WT	TP (°C)
WT	42.7 ± 0.21	---	44.8 ± 0.9
*fad6*	45.0 ± 0.42 **	2.2	44.9 ± 0.5
*act1*	44.2 ± 0.32 **	1.5	44.2 ± 0.7
*fad5*	44.0 ± 0.26 *	1.2	44.2 ± 0.7
*fad7 fad8*	43.8 ± 0.64	1.0	ND
*act1 fad6*	43.0 ± 0.18	0.2	ND
*act1*^++^	43.0 ± 0.53	0.2	ND

**Table 4:** Relative growth rates of Arabidopsis lines at different temperatures. All lines were grown on MS + 1% sucrose media. Growth rate is calculated as the difference of the natural log of seedling dry weight measured over a six d period.

	Rate (ω^-1^)			
	22.5°C	28°C	30°C	34°C
WT	0.52 ± 0.13	0.18	0.22 ± 0.10	0.14
*fad5*	0.34 ± 0.01	0.21	0.24 ± 0.09	0.15
*fad6*	0.37 ± 0.05	0.17	0.24 ± 0.11	0.15
*act1*	0.60 ± 0.24	0.26	0.23 ± 0.02	0.13
*act1 fad6*	0.43 ± 0.15	0.17	0.15 ± 0.04	0.11
*fad7 fad8*	0.51 ± 0.10	0.22	0.26 ± 0.04	0.19
